# Empowering patient choice: a systematic review of decision aids for benign prostatic hyperplasia

**DOI:** 10.1111/bju.16797

**Published:** 2025-05-27

**Authors:** Charlotte Hollands, Debra Gray, Andrew Marren, Richard Hindley, Margaret Husted

**Affiliations:** ^1^ Department of Psychology University of Winchester Hampshire UK; ^2^ Department of Psychology Kingston University London London UK; ^3^ Urology Department Hampshire Hospitals NHS Foundation Trust Winchester UK

**Keywords:** systematic review, BPH, prostate, urology, shared decision making, decision aid

## Abstract

**Background:**

Benign prostatic hyperplasia (BPH) is a complex condition that affects ~3.2 million men in the UK. As men often face multiple treatment options, discussion and consideration of their priorities and preferences is necessary; however, research indicates this is not always adopted in practice.

**Objectives:**

To evaluate decisional interventions currently available for men with symptomatic BPH, distinct from those designed for prostate cancer.

**Methods:**

Eight databases (PubMed, Web of Science, EBSCO, Science Direct, Scopus, Google, Cochrane Library, Centre for Reviews and Dissemination) were searched retrieving a total of 1979 results, of which 13 international studies discussing 10 decision aids (DAs) were included. Studies were eligible that targeted adult males experiencing urological symptoms and discussed any DA designed to promote shared decision making within secondary healthcare.

**Results:**

Narrative synthesis found most DAs focused on treatment information provision; however, risk information was not always equally presented. Most DAs lacked strong theoretical links to existing theories on behaviour change, risk communication, and decision making, and sustained implementation within clinical practice. The most effective aids went beyond information provision, to also elicit and integrate patient preferences and values, by adopting multiple behaviour change techniques (BCTs). Risk of bias indicated medium risk with limited information or justification on data collection and analysis methods.

**Conclusion:**

Current DAs relevant to BPH lack clear focus on individual patient needs required for delivering patient‐centred care. Greater transparency and explicit links to behavioural theory and BCTs related to desired future outcomes, expectations, and values are required to effectively create and implement effective interventions into urological practice.

**Abbreviations**
BCT: behaviour change techniques
DA: decision aid
DVD: digital video disk
IKT: integrated knowledge translation
IPDAS‐SF: short‐form version of the International Patient Decision Aid Standards
MIST: minimally invasive surgical treatment
MMAT: Mixed Methods Appraisal Tool
PICO: Population, Intervention, Comparison, and Outcome
PRISMA(‐S): Preferred Reporting Items for Systematic Reviews and Meta‐analyses (literature search extension)
SDM: shared decision making

## Background

Benign prostatic hyperplasia (BPH) is a common urological condition that affects >210 million men worldwide [1]. The condition is characterised by non‐cancerous growth of the prostate that can have a significant impact on individuals’ urinary function and adverse psychosocial and lifestyle consequences [2]. When attending clinical consultations, patients may face difficulty when making treatment decisions due to the variability of symptoms and diversity of treatment options [1, 3]. With no clear consensus on a single best treatment option, it is imperative that patient preferences and expectations are meaningfully considered during these conversations as part of shared decision making (SDM) [4]. SDM is widely recognised as an important part of healthcare delivery, both internationally [5, 6, 7], within policy in the UK [6], and within specific urological guidance [7, 8, 9, 10, 11]. This approach advocates for healthcare providers and patients to jointly participate in the decision‐making process [12, 13, 14]. This process can be supported using decision aids (DAs) that support treatment discussion and provide critical information to help patients reach a value‐based choice. DAs are meant to be adjuncts to clinicians counselling, so that patients can understand the probable benefits and risk of treatment options, consider the values they place on these, and participate actively with their clinician in selecting treatments that best address their individual needs [15, 16, 17]. In this way, DAs are intended to enrich consultations rather than supplement or replace them, supporting the discussion within the consultation room and facilitating the process of arriving at an informed, values‐based choice [18].

There is mounting evidence supporting the efficacy of DAs in facilitating SDM, reducing decisional conflict, increasing decision quality, and making patients feel clearer about their values [19, 20]. There is currently limited exploration and evaluation of DAs for benign prostatic conditions [21]. There is also limited research on what makes DAs successful in practice. Recent research has begun to use psychological and behavioural theory to address this gap. For example, the behaviour change taxonomy [22] describes different behaviour change techniques (BCTs), which are the smallest components of an intervention that can initiate mechanisms of change [23]. This can then be used to determine why an intervention may be more successful than another, and aid in the development of new interventions or improvement of existing ones. Contemporary research has begun to trial the integration of BCT research with patient DAs to actively to overcome this ‘intention‐behaviour’ gap [24, 25]. However, currently there is limited research in this area, especially for DAs applicable to urological care.

The aim of this study was to understand and evaluate the design, effectiveness, and usage of available DAs in clinical practice for the management and treatment of BPH. For decades, systematic reviews have been hailed as an effective mechanism for informing decisions about improving health care [26]. Therefore, the present review aimed to utilise systematic methods to critically evaluate the existing literature and investigate what DAs are currently available in men's urology, whether behavioural components have been considered in their design, and how their effectiveness in practice has been evaluated and measured. When evaluating the interventions effectiveness, the present review discusses the design, delivery, and mode of the available DAs, considering both patient and clinician experiences, to provide greater insight into the component(s) associated with desirable consultation outcomes.

## Research Questions

- What shared DAs/interventions are currently available within the urological literature that apply to men with symptomatic BPH?
- How have existing DAs/interventions been designed and evaluated within men's urology?

## Methods

The protocol for this systematic review can be found on the International Prospective Register of Systematic Reviews (PROSPERO identifier: CRD42023446600). As stated within this protocol, primary outcomes of interest were intervention characteristic data, including application of theory, frameworks, and BCTs. Secondary outcomes included intervention effectiveness and implementation, including measures such as quality of life (QoL), decisional conflict, adherence, patient satisfaction, knowledge acquisition, and qualitative data on patient's or clinicians’ experiences with the intervention. The review was conducted in accordance with the Preferred Reporting Items for Systematic Reviews and Meta‐analyses (PRISMA) guidelines [27], and all supplementary data files can be found on the Open Science Framework (https://osf.io/b8q92/).

### Search Strategy

The search strategy was initially developed by listing key terms and creating several concept tables and logic grids that captured the key headings according to the Population, Intervention, Comparison, and Outcome (PICO) elements of the review question [28, 29, 30]. Subsequent search strings were developed with the guidance of an academic librarian between October and December 2023, and in accordance with the PRISMA‐literature search extension (PRISMA‐S) checklist [31]. The PICO query was then combined with a narrow filter and adapted according to the output size and relevancy of the first items screened [28].

**Fig. 1:**
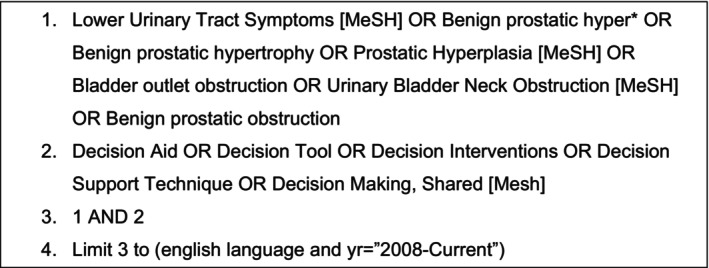
Finalised PubMed search string. MeSH, Medical Subject Headings; yr, year.

In accordance with the Peer Review of Electronic Search Strategies (PRESS) Guideline Evidence‐Based Checklist [32], the review followed an iterative process [33, 34], where combination of terms in their string were pilot tested across two databases, PubMed and Web of Science. The results were then discussed, and strategy finalised with the wider research team, including experts in the fields of both psychology (M.H., D.G.) and men's urological care (R.H.), with previous experience in conducting systematic reviews. Guidance has indicated that for clinical questions the population (P) and intervention (I) are the most frequent elements that need to be addressed [30, 33]. Therefore, the simplified string included keywords and subject headings related to the two main topic areas including: (i) BPH/LUTS/urolog*, and (ii) Shared Decision Making. Figure 1 illustrates the finalised search string for PubMed with all other databases accessible via the Table S1.

### Data Collection

#### Title and Abstract Screening

Using the revised search strategies, searches of the eight databases (PubMed, Web of Science, EBSCO, Science Direct, Scopus, Google, Cochrane Library, Centre for Reviews and Dissemination) were conducted between January and March 2024 by a member of the review team (C.H.). Additionally, the reference lists for included articles were also screened. All retrieved articles from each database were imported into the reference manager Zotero.

In total, 1979 results were retrieved into Zotero and then imported into the on‐line software tool Covidence. After removing 161 duplicates (*n* = 1818), the titles and abstracts of each article were screened independently by three reviewers (C.H., J.K., A.M.). All conflicts were resolved through firstly re‐reviewing the study independently, followed by a discussion between the reviewers and revisiting the inclusion and exclusion criteria (Table 1).

**Table 1:** Inclusion and exclusion criteria.

Inclusion criteria	Exclusion criteria
Randomised controlled trials, evaluation studies, feasibility studies, Quasi‐experimental studies, non‐randomised and qualitative studies	Non‐primary research (i.e., editorials, conference abstracts and presentations). Studies/interventions lacking sufficient publicly available information
Adult males diagnosed or experiencing urological symptoms including but not limited to BPH, and LUTS	Male urological patients aged <18 years or populations made up of only men with prostate cancer
Populations within the UK, Europe and the USA	Populations outside the UK, Europe and the USA
Any decisional intervention designed to generate discussion to help people make choices, by providing information about the options and outcomes, and by clarifying personal preferences and values. The intervention can be delivered in any modality (face‐to‐face or on‐line), and could be implemented at any point within secondary care (i.e., before or after consultation)	Decisional interventions only targeting prostate cancer or delivered within primary care settings
Studies/interventions published between 2008 to 2024	Studies/interventions published prior to 2008

#### Full‐text Screening

Three reviewers (C.H., J.K., A.M.) independently screened 41 studies. Any discrepancies that were identified between reviewers, were resolved through discussion with M.H. At this stage, a total of 13 studies were included for data extraction and evidence synthesis. Reasons for exclusion were categorised into seven explanations inspired by previous work [27, 35]. The most prevalent of these reasons for exclusion was the ‘Wrong Study/Intervention’ (nine studies), followed by ‘Outdated Publication/Data’ (six), and ‘Duplicate Publication/Data’ (five).

A simultaneous search of included articles reference lists identified two additional relevant documents that were subsequently included. The white paper [36] published by the AUA mentioned a patient guide by The Urology Care Foundation [37]. Similarly, a report by the Kings Fund on implementing SDM [38] reported on a pilot programme for SDM within urology departments across different NHS trusts [39].

### Data Extraction

Using Covidence, two reviewers (C.H., A.M.) independently extracted the data, focusing on retrieving the descriptive information of both the studies (i.e., aims, design, participants, and outcomes) and their DAs (i.e., delivery, content, and implementation). Each reviewer also completed the risk‐of‐bias evaluation for the diverse range of included studies (using the Mixed Methods Appraisal Tool [MMAT] [40]), whilst simultaneously evaluating the mentioned DAs (using the short‐form version of the International Patient Decision Aid Standards [IPDAS‐SF]). The MMAT [40] appraises research based on two screening questions (concerning clear and defined research questions/objectives, which are then addressed in the research), followed by five core criteria that are specific to the design of the study. In this way there are differences between the questions for quantitative, qualitative, and mixed‐method studies. Criteria focus on a range of areas including methodological justification/rationale, sampling representativeness, result coherence, interpretation, and integration. Individual outputs by each reviewer were then discussed and compared for any discrepancies in the detail of the response, allowing for a complete consensus on the final reporting of the data.

## Results

**Fig. 2:**
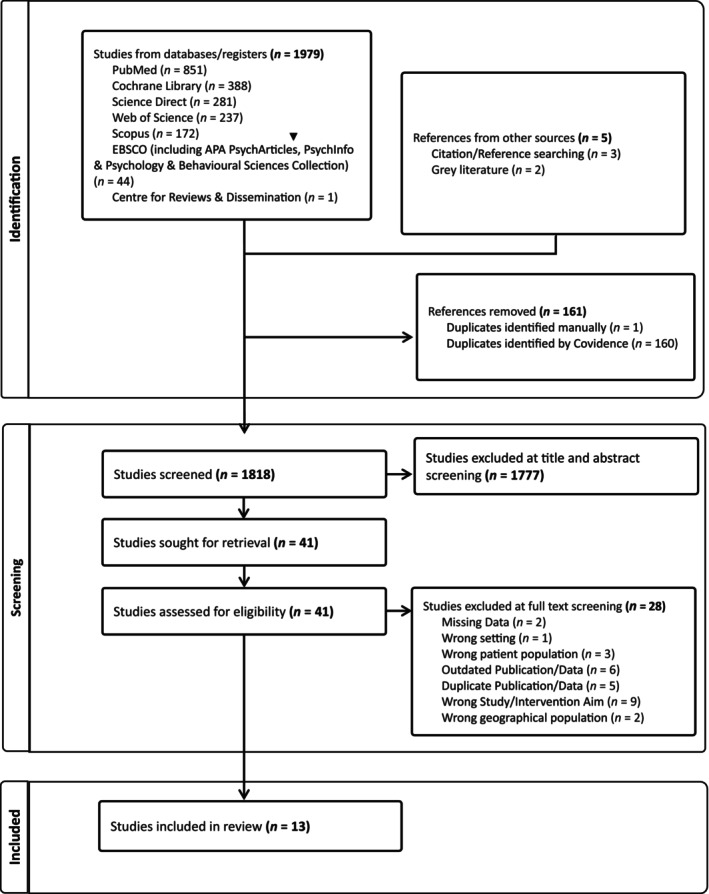
The PRISMA flow diagram displaying the systematic review results at each stage.

The initial search generated 1979 records, after screening 13 articles met the inclusion criteria for this review (Fig. 2).

### Data Analysis

Analysis was undertaken on 13 articles; these articles will be referenced in the text by their corresponding letter shown in Table S1. Of the 13 articles, 10 individual DAs were described. For example, Bouhadana et al. [41, 42] published separate articles on the development and evaluation of the same DA (C, D). Similarly, three separate research articles detailed the process of development, evaluation, and then implementation of the same DA (G, H, M). It was important to retain all the articles despite their overlap, as our objective was to capture how these interventions had been developed, evaluated and implemented. Four interventions were not freely accessible, and therefore only the information included within text, supporting documentation, and diagrams or screengrabs were used for evaluation (B, E, F, K).

### Included Studies

The 13 included studies were conducted across five different countries (UK, USA, Canada, Spain, and the Netherlands). Most studies were categorised as having development methodologies (six studies), closely followed by evaluation (five), and mixed methods (two).

### Included DAs

Of the included 10 DAs, there was an even split in the mode of delivery: four were delivered on‐line through a website, three were delivered as a booklet (accessible on‐line or paper‐based), and three used a combination of different delivery methods including a booklet, digital video disk (DVD), website, and questionnaire. All the interventions were described as patient‐led or self‐administered by the patient themselves.

### Narrative Synthesis

Due to the small sample size and heterogeneity of the studies retrieved, including their methodology and produced interventions, a narrative synthesis was conducted. In this way a textual and iterative approach was adopted to ‘tell the story’ of the findings [43, 44], focusing on the theoretical underpinnings of how the interventions work, why, and for whom, and what enables them to be effective.

#### Developing a Theory

Most of the included studies did not explicitly state a singular theoretical standpoint or model. Whilst there was a general pattern in the conceptual reasoning for why they were designing a DA in this area, to increase patient satisfaction and health‐related outcomes, there was a clear lack of theoretical foundation for the specific components of the interventions. This suggests a muddled narrative for what fundamental mechanisms are being explored and utilised in current DAs, with many seemingly being underpinned by ad hoc knowledge.

When initially discussing or defining SDM, six studies (B, C, D, G, H, M) referenced the Cochrane review of DAs [19, 45], five (A, F, G, H, M) cited seminal research conducted by Elwyn and colleagues [46, 47, 48], and two (K, L) cited AUA guidelines [49, 50]. The remaining four studies referenced patient‐centred care [51] (E), characteristics of SDM [52] (J), men's experiences of BPH treatment decision‐making [53] (I), and the development process of a DA for small renal masses [54] (D). Whilst these references then contained further mentions of relevant models, such as those focused on patient‐centred choice [46, 55], care [56], and communication [57], these were not explicitly discussed or integrated within the included studies. Even those studies that were more descriptive and holistic in their inclusion of different evidence in their systematic/literature review stage (i.e., from existing DAs, treatment outcomes, and clinical guidelines) [41], did not specifically consider and link to existing theories. Some studies described the use of medically established guidelines, such as those produced by the National Institute for Health and Care Excellence, Australian Urology Association, Canadian Urological Association, and European Association of Urology (A, C, D, M), and support from organisations such as the Ottawa Health Research Institute (C, D). Overall, this evidence base is incomplete and unclear, with most DAs lacking clear links between existing theory and the creation of their interventions, and as such it remains difficult to highlight a unifying or anchoring theory that is applicable to all the included DAs.

#### Exploring the Relationship within and between Studies

The heterogeneity between the included studies can largely be attributed to the differences in study design and methodology. Most studies were conducted within the USA (B, E, F, K, L), followed by the Netherlands (G, H, M), UK (A, I), Canada (C, D) and Spain (J), and primarily focused on hospital settings in secondary and tertiary care.

The development processes described by studies varied from basic iteration cycles of a single consultation with stakeholders, to more complex processes, with rounds of alpha testing with patients and subsequent beta testing within clinical practice. Within studies with larger samples, they looked beyond patients and consultants to also consult nurses, charities, and policy advisors (A), whereas others focused more on creating steering committees of patient advocates, clinical, and methodological experts (C). However, the review identified a lack of clear and detailed reasoning justifying their chosen research design and methodology.

Measurements for the effectiveness of the DAs varied between studies. They ranged from focusing on total healthcare cost and the rate of BPH surgery 6 months after DA use [58] (B), to assessing patient knowledge and experience of DA use through qualitative interviewing (F), and patient's self‐reported treatment preference before and after DA use (H), or between DA users and a usual care group [59] (K). Of the 13 papers, seven addressed or discussed DA effectiveness or feasibility (B, D, E, F, H, K, M), with only one study (M) demonstrating effectiveness beyond patient knowledge or matched treatment, to outcomes including decisional conflict, process regret, and experience of SDM. This lack of a clear, reliable, and consistent measure of efficacy across studies means the primary purpose of the DAs (to support the process of patient decision‐making) is not established.

Additionally, common barriers to implementation from a practitioner and broader healthcare system perspective were discussed including time constraints, concerns for fitting the DA within existing workflow, organisational support, and cost were mentioned (B, E, H). However, only one study was identified that described a form of training for physicians associated with their intervention [60] (J), and two described a process of adherence measurement to ensure the intervention was being used as intended (F, M). Without training, the DA may not be understood or implemented as intended. The lack of adherence measures puts into consideration the validity of the effectiveness measure being used, as the implementation of the DAs is not being tracked or ensured by specific and detailed intervention procedures. Where provided, the details on if this training specifically addressed and tackled barriers to implementation were unclear.

In conclusion, there was significant variation in both the development phases and evaluation criteria of the interventions. Similarly, sustained implementation within urological practice was limited, with a lack of discussion on the use and effect of training or engagement strategies with staff and patients on the uptake and effectiveness of DAs.

#### Similarities and Differences between Interventions

There was significant overlap in the information on the benefits and risks for specific treatment options. Most interventions mentioned the use of medication (including α‐blockers, inhibitors and a combined approach), surgical options such as a prostatectomy, TURP, and sometimes also watchful waiting and lifestyle changes. More recently developed DAs also discussed minimally invasive surgical treatments (MISTs) including laser, water vapour, Aquablation, Rezum, UroLift, and prostatic urethral lift (D, H, I, L). However, the presentation of these risks and benefits varied considerably from textual descriptions and natural frequencies to more visual representations using diagrams and charts. This is likely due to the interventions drawing on a range of different datasets and publications for these figures and statistics from mainly the Organization for Economic Cooperation and Development (OECD) and Western countries. Furthermore, this variation emphasises how current DAs within the field are largely dependent on current medical guidelines and available research studies, particularly in regard to newer treatments such as the expanding range of MIST options. This rapidly changing treatment landscape creates a unique situation for DA development, whereby continual refinement is necessary to ensure that provided treatment information remains up‐to‐date with research findings on efficacy and side effects.

Beyond the starting content of health information, the interventions greatly differed in their approaches to create personalisation and elicit individual preferences. For example, some interventions encouraged patients to complete the IPSS questionnaire (H, L) or select their prostate size (D). Others encouraged the completion of a personal decision form (A), or produced a personalised patient report (K), based on their prostate size, prior prostate surgeries, past and current BPH medications, and a ranking of patient preferences for outcomes. One intervention specifically asked patient's what is most important to them using three questions, with a further two pages dedicated to preparing them for their next appointment and encouraging patients to think about potential questions they may want to ask in the future (I). Taking this process further, two DAs (C, G) required patients to complete several rounds of value clarification exercises using rating scales to match patients’ preferences for certain outcomes to the most appropriate treatment option. Future DAs should consider the importance of including specific elements designed to appropriately elicit patient preferences, in conjunction with their current healthcare and lifestyle status. This information would need to be provided in a comprehensive and succinct format that summarises what is most important for the patient to then be discussed with healthcare professionals and loved ones regarding men's treatment plans and goals.

Interventions were delivered using a combination of different modalities (i.e., printed leaflets, website, DVD), with one study (F) finding that men's preferences for the mode of their DA were shaped by the order of the information, the stage of their condition, and stage of treatment decision‐making. Only one intervention was routinely implemented and evaluated across outpatient departments within five Dutch hospitals (M); however, the DA delivery was not standardised between hospitals. There remains a lack of clear consensus on the best way to deliver these interventions within existing urological practice, highlighting a potential barrier to future implementation. This is a concern as the evidence would indicate that current research on the appropriateness and efficacy of DAs applicable to BPH has often not moved beyond feasibility trial stages.

In conclusion, whilst there was significant overlap in the treatment information given across the DAs, they varied in the presentation of associated risks and benefits. Similarly, the delivery of interventions varied, and sustained implementation within practice was limited or unstandardised. Furthermore, the interventions differed in their approaches to eliciting patients’ preferences, with only a few adequately describing any reflective activities, and even fewer matching these to the most appropriate treatment option(s).

### Components of Effective of DAs

The most highly rated interventions (based on the IPDAS‐SF) were those that included BCTs that focus on individuals desired future outcomes, expectations and values, comparison of outcomes and valued self‐identity. Seven different BCTs were identified across the interventions including information about health consequences, salience of consequences, information about others approval, pros and cons, comparative imaging of future outcomes, and valued self‐identity (5.1, 5.2, 6.3, 9.1, 9.2, 9.3, 13.4). However, three studies (B, E, K) did not provide sufficient information or accessibility to their interventions to be able to adequately identify individual BCTs. On average, three BCTs were evident across all other evaluated interventions, with BCTs related to information about health consequences and pros and cons (5.1 and 9.2) the most common.

The most effective DAs went beyond information provision on the condition and the benefits and risks of treatment. What distinguished the top four performing interventions (A, C, G, I) was their inclusion of specific patient needs and expectations during the development phase. The best scoring DAs (C, G) built on this by eliciting reflections on the personalised information to integrate these values into the recommended treatment. It should be acknowledged that two additional studies still reported positive patient and practitioner feedback, despite scoring lower (D, J). For these two studies the patients who assessed these DAs had moderate symptom severity (IPSS mean [sd] 11.16 [8.47]) and prostate size (30–80 mL), which may have influenced acceptability positively. However, full demographic patient data for these evaluating groups (D, J) in terms of race, ethnicity, socioeconomic status, and education were not always clearly reported or discussed.

In terms of evaluated effectiveness in practice, five studies (A, B, H, K, M) discussed or described the implementation of their DAs within various settings, including a multi‐site group practice in primary care (B), as well as secondary (A, H, M) and tertiary care services (K), all within the urology speciality. Whilst three other studies (D, E, F) described the evaluation of their DAs, this was in the context of feasibility. In this way, the DAs were assessed on their suitability (i.e., behavioural intention to use in practice by consultants or patients’ preference for modality), and further assessment of its use within clinical practice were not conducted. This is an important distinction for research as intervention studies can go further to bridge the gap between research and practice. Further research is necessary to evidence how different practices and engagement strategies with staff/patients effect the uptake and effectiveness of DAs.

To summarise, the review findings indicate that effective interventions go beyond basic information provision to include a range of BCTs that focus on individuals desired future outcomes, expectations and values, and provide the opportunity to compare and evaluate outcomes according to the different options. However, future research evaluating DAs in practice must go beyond feasibility to assess efficacy and use within clinical settings.

### Risk of Bias

Risk‐of‐bias evaluation using the MMAT [40] focused on appraising each included article based on two screening questions, followed by five core criteria that coincided with the article's methodological category. The overall risk‐of‐bias judgements were determined by firstly assessing the ratings of risk for individual criteria, and then collectively assessing risk across multiple domains (Table 2 [20, 21, 37, 39, 41, 42, 51, 57, 58, 59, 60, 61]). Findings highlighted how seven (A, C, D, E, F, G, I) of the 13 included articles did not clearly state a research question. Three (A, C, I) of these seven studies also provided limited information, detail or, justification on their data collection methods. Most studies were classified as mixed methods (six studies), this incorporated many of the different development and evaluation studies as they applied both quantitative and qualitative techniques. Four of these studies (A, C, D, J) lacked detail or did not report on how inconsistencies were addressed, or the integration of methods and data, an especially important element of development studies. The importance of including different stakeholders within this process was described by some articles (A, C, G, I, J), but it was often unclear how this information related to each other, which similarities and differences were identified, integrated, and resolved, and which elements ultimately made up the resultant DA.

**Table 2:** Evaluation of included studies using the MMAT.

Publication	A.	B.	C.	D.	E.	F.	G.	H.	I.	J.	K.	L.	M.
	Archer and Finn, 2011 [39]	Arterburn et al., 2015 [58]	Bouhadana et al., 2021 [41]	Bouhadana et al., 2021 [42]	Chhatre et al., 2021 [51]	Halley et al., 2015 [57]	Lamers et al., 2016 [61]	Lamers et al., 2020 [21]	NHS England., 2024 [62	Perestelo‐Perez et al., 2010 [60]	Sadik et al., 2021 [59]	Urology Care Foundation, 2023 [37]	Van der Wijden et al., 2019 [20]
MMAT Questions													
	S1	S1	S1	S1	S1	S1	S1	S1	S1	S1	S1	S1	S1
	S2	S2	S2	S2	S2	S2	S2	S2	S2	S2	S2	S2	S2
	Q5.1	Q3.1	Q5.1	Q5.1	Q5.1	Q5.1	Q3.1	Q4.1	Q1.1	Q5.1	Q3.1	Q4.1	Q3.1
	Q5.2	Q3.2	Q5.2	Q5.2	Q5.2	Q5.2	Q3.2	Q4.2	Q1.2	Q5.2	Q3.2	Q4.2	Q3.2
	Q5.3	Q3.3	Q5.3	Q5.3	Q5.3	Q5.3	Q3.3	Q4.3	Q1.3	Q5.3	Q3.3	Q4.3	Q3.3
	Q5.4	Q3.4	Q5.4	Q5.4	Q5.4	Q5.4	Q3.4	Q4.4	Q1.4	Q5.4	Q3.4	Q4.4	Q3.4
	Q5.5	Q3.5	Q5.5	Q5.5	Q5.5	Q5.5	Q3.5	Q4.5	Q1.5	Q5.5	Q3.5	Q4.5	Q3.5

Key	
	Yes
	Cannot Tell
	No

Each study evaluated by two screening questions and a further five core criteria based on their study design. Overall medium to high risk‐of‐bias judgements determined by high risk in at least one domain (No) or some concerns (Cannot Tell) for multiple domains [63]. Further detailed explanation can be found within the supplementary material (https://osf.io/b8q92/).

## Discussion

This systematic review identified 10 DAs, across 13 international studies, applicable to men with BPH. Six studies were identified as at medium‐to‐high risk of bias due methodological and analytical limitations. Many DAs lacked connections to existing behavioural theory and sustained implementation within practice. The most effective interventions focused on selecting and integrating patient preferences.

### Theoretical Frameworks in the Development of DAs

A strength of some studies was the involvement of multiple key stakeholders; however, it remains unclear how stakeholders are informing the development of DAs. Importantly, DAs are expected to be user‐tested, open to scrutiny, and well‐documented [64], this study found that, consistent with other reviews [65, 66], few articles reviewed adequately described development methods. Given that DAs require engagement from both patients and practitioners, elements that explore users’ needs and clinical workflows are key and could increase future use in practice [67]. This is important because studies that did have higher quality scores, such as Bouhadana et al. [41], report patients and urologists attributed different importance to specific outcomes for treatment decision‐making. For example, patients placed greater emphasis on functional outcomes, such as time to return to work, in comparison to urologists who were more concerned with clinical outcomes, such as symptom improvement. Addressing these differences is needed to improve clinical consultations and the decision‐making processes. Despite this, most other studies did not report on similarities or divergences between the needs of different stakeholders, or how these needs were integrated together to develop a finalised DA. This represents a core limitation, as it was not clear which pieces of data were being integrated into components of the DA. Therefore, it is difficult to identify how differing views, or evidence, may have been prioritised to create the intervention and who it ultimately serves.

Whilst some studies acknowledged models of SDM, many of them did not explicitly state a singular theoretical standpoint or link existing theory to the creation of their interventions. This in turn led to a reliance on collecting data from a specific set of stakeholders and effected the evaluation process, where detailed demographic data for these groups was often lacking or unclear. Underpinning theories are essential to help improve understanding of the underlying mechanisms driving behavioural change within interventions. Models or theories can provide guidance on the design and clarity on which components should be evaluated and refined to create effective interventions [68].

Intervention studies can bridge the gap between research and practice, and this remains a key missing next step for many DAs designed for men with BPH. To successfully achieve this, it would be beneficial to initially adopt a multidisciplinary perspective and integrate existing psychological knowledge on the process of SDM, patient experience, cognitive decision‐making, value clarification, and behaviour change with the clinical symptomology and medical expertise from clinicians and existing medical guidelines. This has been suggested previously [69], whereby to support implementation and continued use of DAs, researchers could involve stakeholders from third sector organisations along with users and health professionals in an integrated knowledge translation (IKT) approach [69, 70, 71]. As an approach to research, IKT has the potential to ensure that research knowledge does, in fact, respond to identified decision‐maker needs [70, 72]. A psychologically informed approach would allow these interventions to be designed with individual differences in behaviour and future implementation in mind, so that components of the DA can be appropriately refined and tested.

### Importance of Patient Preferences in the Content and Delivery of DAs

The review indicates DAs only, or primarily, concerned with information provision are of poorer quality and efficacy. Future DAs should engage with a wider breadth of BCTs. Multi‐component DAs have been shown to be preferable in other areas of medicine and patient decision‐making [73, 74, 75, 76]. However, importantly, most studies did not describe specific theoretical underpinnings for the development process of their intervention or make specific reference to BCTs [22], a concern which has been linked to the limited effectiveness of interventions within healthcare contexts [77, 78]. Theory‐informed interventions are beneficial and necessary because their overall impact and effectiveness can be assessed using existing frameworks for structured evaluation. This cycle allows for continuous improvement by providing insight into what specifically makes some interventions more successful than others. This is especially important within healthcare, as endorsed by The Medical Research Council that emphasises the importance of integrating theory and the best available evidence to develop complex interventions [79]. However, it should be noted that the aim of the current interventions within urology is not necessarily to change patient's behaviour but rather support them through the treatment decision‐making process. Therefore, it cannot be assumed from our results that the specific BCTs identified represent the entirety of why an intervention was successful or effective or not.

The DAs themselves were similar, particularly in terms of their content and structure. However, treatment information was not always equally presented for negative and positive elements, with many studies also using different presentation methods, e.g., visual diagrams, graphs, or textual descriptions in natural frequencies (i.e., one in 100 men). The way that statistical and healthcare‐related information is framed can have a profound impact on our perception and decision making [80]. Numeracy skills are robustly related to accurate perceptions of health benefits and risks, the quality of SDM, and health outcomes [81, 82]. Patients can find it difficult to understand numerical representations like ratios and probabilities [83], due to lower numeracy skills [84] and cognitive biases. These include the denominator neglect effect, whereby individuals focus too much on the negative aspect in a ratio and insufficient attention paid to the context/denominator [82]. There is also evidence that some visual formats like icon arrays/pictograms are more effective than others (e.g., pie charts) for representing risk [85]. For men with BPH, treatment decisions are often made under uncertainty, and therefore it is key to consider the effect of risk framing when developing DAs.

Lower overall scores for the worst performing DAs using the IPDAS‐SF were attributed to poorer individual scores on the dimensions of decision support tool evaluation, development, and specifically patient needs and values, including personal importance. In accordance with previous reviews into prostate cancer [66], a minority of DAs contained interactive methods to identify patients’ values and preferences, or to compare pros and cons of the available options. The analysis also showed that DAs with the highest quality score adopted multiple BCTs, including natural consequences, associations, comparison of outcomes and identity. For example, the highest scoring intervention by Lamers et al. [61] included treatment information, healthcare consequences, as well as advising patients to rate and compare their most valued outcomes and then compare these with what different treatment options. This was supported by 79% of patients receiving the treatment that they had indicated as their preference after DA use [20]. What distinguished the higher rated four DAs was their inclusion of specific patient needs during the development phase, and the inclusion of BCTs related to prompting individuals to consider their desired future outcomes, expectations, and values. This supports previous research that advocates how DAs must address not only medical factors (e.g., recovery, side effects), but also personal or lifestyle factors, such as how they weigh the risks and possible benefits of treatments [86]. This is especially important for a condition such as BPH where management and treatment require preference‐sensitive decisions as there is no clear consensus on a single best treatment option, meaning patient preferences need to be highlighted, discussed, and meaningfully considered during patient and clinician conversations [53, 66].

### Implementation within Clinical Practice

Three studies discussed the feasibility of their intervention (D, E, F), but this was largely restricted to a focus on the acceptability of the DA among key stakeholders including patients and consultants. Whilst five other studies described some form of clinical implementation, many lacked clear reporting or further details on the process including any barriers or facilitators with staff, patients, or the organisational structure of the healthcare setting. This issue may be partially due to many studies advocating for the use of their DAs prior to medical consultation; however, with a lack of adherence measures, many did not address whether patients who received DAs viewed or used them. Similarly, there was no discussion on whether the conversations between patients and providers changed as result of implementing these DAs. Within this area of urology, studies designing DAs must now go beyond feasibility and behavioural intention, to look towards assessing actual use within clinical practice. As such, future studies need to actively track, monitor, and evaluate any system‐level changes to better understand how the differences between methods of implementation influence the uptake and effectiveness of DAs.

## Conclusion

This review evaluated the design, effectiveness, and usage of available DAs for the management and treatment of BPH. Despite some DAs lacking accessibility and clear theoretical foundations, there is evidence to suggest further development of the interventions within this field has the potential to improve the process of SDM for patients in urological practice. The most effective DAs were identified as ones that went beyond basic information provision, to elicit and integrate patient preferences and values into the recommended treatment and discussions with healthcare professionals. The analysis clearly identified both methodological and theoretical limitations. Therefore, future research of DAs within men's urological care should follow more transparent and detailed reporting, meaningfully incorporate key psychological theory on decision making, and be tested within the relevant clinical context [87, 88].

## Plagiarism Check

The author(s) confirm that the manuscript has not been previously published and is not under consideration elsewhere and accept that our manuscript may be screened for plagiarism against previously published work.

## Disclosure of Interests

The author(s) declare no potential conflicts of interest with respect to the research, authorship, and/or publication of this article.

## Funding Statement

Sir James Aurthur Ratcliffe has funded a studentship, which has facilitated the undertaking of this research as part of the wider Optimising Patients’ surgical Treatment choices In Male benIgn prostatic hyperplasia (OPTIMISE) project (https://osf.io/378a2/).

## Ethics Statement

This systematic review was conducted in accordance with the PRISMA statement. As this study involved the analysis of publicly available data and did not involve direct interaction with human participants, ethical approval was not required. All data were obtained from publicly accessible databases and published articles, ensuring compliance with the ethical standards of secondary data usage.

## Supporting information


**Table S1.** Summary of characteristics of articles included in the systematic review (*n* = 13).

## Data Availability

All supplementary data files are available on the Open Science Framework: https://osf.io/b8q92/.
