# Supplementary material for: Empowering patient choice: a systematic review of decision aids for benign prostatic hyperplasia
Source: BJU Int. 2025 May 27;136(3):359–71. doi: 10.1111/bju.16797 (PMC12343979; doi:10.1111/bju.16797)
Supplement: Supplementary file 1 — Table S1. Summary of characteristics of articles included in the systematic review (n = 13). [file BJU-136-359-s001.docx]

**Table S1.** *Summary of characteristics of articles included in systematic review (n = 13)*

| **Study Characteristics** |  |  |  | **Intervention Characteristics** |  | **Results** |  |
| --- | --- | --- | --- | --- | --- | --- | --- |
| **Reference**  **First Author (year)** | **Setting/Context** | **Participants/Sample** | **Methodology/Study Design** | **Target Population** | **Intervention** | **Main Findings** |  |
| 1. Archer & Finn (2011) | NHS Foundation Trust Hospitals,  Secondary care urology services in England, Wales, and Scotland (n=11). | Experts, academics, consultant urologists, BPH and uro-oncology specialist nurses, government officials, individuals from healthcare organisations, patient representatives, and urology focused charities (n=63). | Development Study | Men diagnosed with LUTS caused by an enlarged prostate. | English Language  39-page Booklet and DVD  Implement Before Consultation | Clinicians saw the value in the DA as a way to inform patients and allow for a more individualised discussion during the consultation to focus on patient options.  Patients and their partners noted having time to explore options and information, reflect without time pressure.  The DA was developed for patients with a reading age of 12 years old and granted the Plain English Campaign Crystal Mark. |  |
| 1. Arterburn et al (2015) | Group Health Urology Services, healthcare within Washington State. | Patients with BPH or LPC > 45 years in contact with the Group Health Urology Services (n= 554). | Evaluation/Feasibility Study | Patients diagnosed with BPH | American English Language  Video (DVD/online) and Leaflet  Implement Before Consultation | DA use was associated with a significant 32% lower rate of TURP among men who had previously received pharmacological treatment for BPH.  Among previously untreated patients, receiving a DA was associated with a non-significantly higher 180-day rate of TURP procedures.  DAs use lowered use of elective surgery, at least in the short term. |  |
| 1. Bouhadana et al (2021a) | Systematic Review | Systematic Review (n=41 included articles), and a steering committee of BPH patient advocates (n=2), methodological experts (n=2), and clinical experts that were either community or academic-based urologists (n=6). | Systematic Review/Development Study | Patients diagnosed with LUTS secondary to BPH | Canadian English Language  Interactive Online Information and Questionnaire.  Implementation not specified; however, patients encouraged to discuss the printable summary with their doctor at their next consultation. | Patients emphasised functional and relatable outcomes and were less interested in quantitative outcomes if not correlated to concrete outcomes. For example, patients were interested in outcomes such as time to return to work. Conversely, urologists were more concerned with traditionally reported outcomes such as IPSS improvement.  The readability of the DA had a SMOG score of 8.4. |  |
| 1. Bouhadana et al (2021b) | Urologists recruited from both academic and community settings. | Patients (n=19) who had received a surgical treatment for their BPH, and urologists who treat BPH surgically (n=11). | Evaluation/Feasibility Study | Men deciding a surgical treatment option for LUTS/BPH. | Canadian English Language  Interactive Online Information and Questionnaire.  Implementation not specified; however, patients encouraged to discuss the printable summary with their doctor at their next consultation. | Patients agreed that the language used in the DA was easy to follow (100%), the amount of information provided was adequate (84.2%), the length was appropriate (84.2%), the outcomes were easy to understand (89.4%) and indicated that they would recommend it to new BPH patients (100%).  Patients vocalised the benefits of combining physician expertise with the evidence-based and structured framework used in the DA. |  |
| 1. Chhatre et al (2021) | Large urban academic medical centre | Patients with OAB (n=62) and urological providers (n=16). | Mixed Methods Study | Patients with OAB | American English Language  Web-based tool (OABCare)  Implement Before Consultation | SR (n=49) identified barriers  including invasiveness, embarrassment, time constraint, and cost.  Top five attributes were identified as concerns about caregiver burden, impaired bladder function, social interaction, side effects, and use of pads.  The pilot study found 94% of patients found the definitions easy to understand, and 86% felt it was helpful in deciding treatment. |  |
| 1. Halley et al (2015) | Large multi-speciality ambulatory care setting | Patients with Colorectal Cancer (n=30), Diabetes (n=30) or BPH (n =30). | Mixed Methods Study | Patients diagnosed with either Colorectal Cancer, Diabetes or BPH | American English Language  DVD with booklet, and interactive website  Implementation not specified | 44% of patients preferred web-based DA,  32% the DVD, and 24% said both. Those who viewed the Web first, accessed 2x more interactive elements than people who viewed the DVD first.  For BPH group, there was a significant interaction between randomisation group and knowledge over time (F (2,27) = 4.61, *p* = 0.019). DVD-format was more effective for increasing knowledge than the Web-based tool.  Preferences shaped by information structure and stage of the condition/decision. |  |
| 1. Lamers et al (2016) | Dutch hospitals in the southern part of the Netherlands. | Urologists (n=15),  patients with BPH (n=26),  Urology Resident (n=1) Psychologists (n=2), Expert in medical decision making (n=1), and a policy adviser (n=1). | Development Study  (Four stage Delphi consensus) | Patients diagnosed with LUTS/BPH by a urologist. | Dutch Language (translated)  Interactive Web-based information and questionnaire (values clarification)  Recommended to implement before consultation | A consensus was reached on  statements for  decision options, structure, and medical content for 61% of urologists, and on clarifying patients’ preferences for 69% of patients.  Used statements to create a web-based DA.  Pros of the DA identified by patients were clear information provision, balanced, systematic design and easy to read and re-read (N = 10). |  |
| 1. Lamers et al (2020) | Dutch hospitals in the southern part of the Netherlands. | Patients diagnosed with LUTS/BPH (n=126). | Evaluation/Feasibility Study | Patients diagnosed with LUTS/BPH making a treatment choice. | Dutch Language  (translated)  Interactive Web-based information and questionnaire (values clarification)  Recommended to implement before consultation | 65% of patients were able to indicate a preferred treatment after using the DA (compared to 47% clear preference before DA). Those patients who did not have an initial preference, were able to make one after DA use (51%).  80% of patients who did have an initial treatment preference remained the same after using the DA.  Most VCEs were discriminative between final treatment preferences and congruent with final treatment preferences, however VCE for side effects from medication & lifestyle change were not discriminate.  For 79% of patients, their preferred treatment  matched their received treatment, however 21% patients did not.  70% of healthcare professionals said they would recommend it to their colleagues, and 69% would want to continue using it in the future. |  |
| 1. NHS England (2024) | Primary and secondary care within the NHS | Patient testers (n=21, 9 with a BPH diagnosis, 11 without a diagnosis, and 1 equality & inclusion professional), Expert advisory group (n=12), and Clinicians (n=4). | Development Study* | Men with an enlarged prostate/BPH. | English Language  Booklet (accessible online and printable)  Implement Before Consultation | Developed a patient DA for men with BPH in the form of a booklet which can be used across primary and secondary care within the NHS. |  |
| 1. Perestelo-Perez et al (2010) | Urodynamics and Urology Services of the Hospital Universitario de Canarias (HUC), Tenerife, primary healthcare centres as part of the Spanish National Health System. | Phase 1, Systematic Review (n=18 papers)  Phase 2, Panel of researchers and clinicians, and focus group of male patients (n= 20)  Phase 3, Patients with hip or knee osteoarthritis (OA) (n=133) | Development Study | Patients with either hip or knee OA, BPH, or depression. | Spanish Language  (translated)  Paper-based booklet  Implementation not specified; however, recommended that they should discuss their options with their doctor. | Men from the focus group stated the DA provided an adequate amount of information (95%), the information was understandable (75%), it helped them think of new questions to ask their doctor (75%), and they would recommend it to another man with BPH (100%). |  |
| 1. Sadik et al (2021) | Single academic tertiary care centre. | Patients between 18-85 years with an ICD-10 diagnosis code for BPH (n=255) and  Urologists (n=10). | Evaluation/Feasibility Study | New patients to the care centre diagnosed with BPH. | American English Language  Online information and interactive website.  Implement Before Consultation | DA use was associated with a significant decrease in procedural management of BPH, even after controlling for BPH medication status (usual care 2.6 times more likely).  A high proportion of patients were evaluated by urologists without exhausting primary care management options. |  |
| 1. Urology Care Foundation (2023) | Systematic Review conducted by Minnesota  Evidence Review Team. | Men diagnosed with LUTS attributed to BPH. | Systematic Review/Development Study | Men diagnosed with LUTS/BPH. | American English Language  Booklet (accessible online and printable)  Implement Before Consultation | AUA produced 43 guideline statements for practice including how patients should be counselled on options for intervention. These clinical principles and expert opinion were then used by UCF to develop the DA. |  |
| 1. Van der Wijden et al (2019) | Outpatient clinics in Dutch hospitals, Netherlands. | New male patients with LUTS/BPH who consulted the urologist (DA group, n=109, control group, n=108) | Evaluation/Feasibility Study | Men diagnosed with LUTS/BPH. | Dutch Language  (translated)  Interactive Web-based information and questionnaire (values clarification)  Recommended to implement before consultation | DA group had lower decisional conflict and process regret (other aspects of regret did not differ between the groups), felt more informed, and clearer about their values.  Involvement in SDM was slightly higher in the DA group than in the control.    By improving patient knowledge, patients were more likely to choose conservative treatments if they had not used prior medication. |  |

***Note:*** *supporting document detailing development of a published decision aid, rather than a standalone published research article

LUTS = lower urinary tracts symptoms, BPH = benign prostatic hyperplasia, OAB = overactive bladder, LPC = localised prostate cancer, OA = osteoarthritis, SR = systematic review, SMOG = Simple Measure of Gobbledygook, TURP = transurethral resection of the prostate, IPSS = international prostate symptom score
